# Metal-Decorated C_8_ Quantum Dots as Lightweight Hydrogen Storage Materials: A Comprehensive DFT Study

**DOI:** 10.3390/nano16050286

**Published:** 2026-02-24

**Authors:** Seyfeddine Rahali, Ridha Ben Said, Youghourta Belhocine, Suzan Makawi, Bakheit Mustafa

**Affiliations:** 1 Department of Chemistry, College of Science, Qassim University, Buraydah 51452, Saudi Arabia; r.said@qu.edu.sa (R.B.S.); s.alkhaleefa@qu.edu.sa (S.M.);; 2 Laboratory of Catalysis, Bioprocess and Environment, Department of Process Engineering, Faculty of Technology, University of 20 August 1955, Skikda 21000, Algeria; y.belhocine@univ-skikda.dz

**Keywords:** hydrogen storage, C_8_ quantum dots, metal decoration, density functional theory, reversible adsorption, thermodynamic analysis, gravimetric capacity

## Abstract

Lightweight, efficient, and reversible hydrogen storage materials are critical for the advancement of hydrogen-based energy technologies. In this work, we present a comprehensive density functional theory (DFT) investigation of hydrogen storage in pristine and metal-decorated C_8_ carbon quantum dots (CQDs), representing ultrasmall, highly curved nanomaterials at the molecular–nanoscale interface. Lithium, magnesium, and titanium were investigated as representative decorating metals to tailor hydrogen adsorption strength and reversibility. The pristine C_8_ quantum dot is structurally stable but exhibits negligible hydrogen affinity (−0.062 eV per H_2_), rendering it unsuitable for practical storage applications. In contrast, metal decoration significantly enhances hydrogen adsorption while preserving molecular H_2_ physisorption, yielding optimal single-molecule adsorption energies of −0.172, −0.304, and −0.451 eV for Li-, Mg-, and Ti-CQDs, respectively. Sequential adsorption analysis indicates exceptionally high hydrogen uptakes of up to 18 H_2_ molecules for Li-CQD and 20 H_2_ molecules for both Mg- and Ti-CQDs, corresponding to very high theoretical gravimetric capacities. Energy decomposition and interaction region analyses demonstrate that hydrogen uptake proceeds via a cooperative physisorption mechanism driven by dispersion, electrostatic, and polarization interactions, strongly enhanced by quantum confinement and extreme curvature effects inherent to the CQD. Grand canonical thermodynamic modeling confirms fully reversible hydrogen storage under practical temperature and pressure conditions. Among the systems studied, Mg-CQD exhibits the most favorable balance between adsorption strength and desorption accessibility, delivering a remarkable reversible gravimetric hydrogen storage capacity of 21.7 wt%, significantly surpassing most metal-decorated graphene-, fullerene-, and carbon nanotube-based materials reported to date. These results establish metal-decorated C_8_ quantum dots as a new class of high-performance nanomaterials for reversible hydrogen storage and demonstrate the potential of ultrasmall carbon quantum dots to overcome the long-standing trade-off between hydrogen uptake and reversibility in nanostructured storage media.

## 1. Introduction

The transition toward a sustainable and carbon-neutral energy economy urgently demands efficient, safe, and high-density energy carriers [1,2]. Hydrogen, with its high gravimetric energy density and zero-carbon emission upon combustion, stands out as a promising candidate to replace fossil fuels in transportation and stationary applications [3]. However, the widespread adoption of hydrogen technology is critically hindered by the lack of materials capable of storing hydrogen with high capacity, fast kinetics, and full reversibility under near-ambient temperature and pressure conditions [4,5].

Conventional storage methods, such as high-pressure compression and cryogenic liquefaction, suffer from significant safety risks, high energy penalties, and low volumetric efficiency [6,7]. Solid-state storage via physisorption or chemisorption in porous nanomaterials offers a promising alternative, provided the adsorbent exhibits an optimal binding energy window (approximately −0.10 to −0.60 eV per H_2_ molecule) to ensure easy charging and discharging [8,9]. To meet the stringent gravimetric targets set by the U.S. Department of Energy (DOE), materials must be composed of lightweight elements, thereby maximizing the weight percentage of stored hydrogen [10,11].

In this context, carbon-based nanostructures have been extensively investigated due to their low atomic mass, high surface area, and tunable electronic properties [12]. Research on carbon nanomaterials for hydrogen storage has evolved from classic structures such as graphene, fullerenes (C_60_), and nanotubes to more complex and tunable systems such as graphene quantum dots (GQDs) and carbon nanoclusters [13,14,15]. Among these, small carbon clusters (C_n_) serve as fundamental building blocks and model systems to understand the interplay between size, curvature, electronic structure, and adsorption energetics [16]. The C_8_ cluster, in particular, has attracted attention for its unique geometry, high stability, and significant curvature, which can induce charge polarization and create active sites for molecular adsorption [17]. Recent DFT studies have shown that C_8_ nanocages exhibit remarkable adsorption strength for heavy metals such as chromium atoms, with adsorption energies as high as −8.74 eV, accompanied by significant charge transfer, structural deformation, and a reduction in the energy gap [18]. These findings highlight the inherent reactivity of C_8_ and its ability to form strong, partially covalent interactions with adsorbates, a property that can be strategically harnessed for hydrogen storage through targeted metal decoration.

Unlike extended graphene sheets, quantum-confined systems like C_8_ exhibit discrete electronic states and enhanced surface reactivity, making them intriguing candidates for host–guest interactions [19]. Despite its fundamental interest and demonstrated adsorption capabilities, the potential of the pristine C_8_ quantum dot as a hydrogen storage medium remains largely unexplored. More importantly, while metal decoration is a well-established strategy to enhance H_2_ binding in carbon nanostructures through charge-induced polarization (alkali metals), orbital hybridization (transition metals), or electrostatic interactions (alkaline earth metals), its systematic application to the C_8_ cluster has not been reported [20]. For instance, Li decoration on graphene can improve H_2_ adsorption to ~0.2 eV, while Ti decoration can induce stronger binding (~0.4–0.6 eV) via d-orbital coupling [21,22]. Magnesium, an alkaline earth metal, offers a promising compromise between light weight, cost, and its ability to polarize H_2_ molecules through charge transfer, yet its performance on curved, low-dimensional carbon systems like C_8_ is not well understood [23].

Computational modeling based on density functional theory (DFT) has become a powerful tool for the investigation of hydrogen storage materials [24,25]. First-principles calculations allow efficient screening of structural stability, adsorption energetics, gravimetric capacity, and reversibility prior to experimental validation [26,27,28]. In hydrogen storage research, computational approaches are particularly valuable for identifying optimal adsorption energy ranges and practical operating conditions. Moreover, atomistic simulations provide insight into structure–property relationships and guide the rational design of lightweight and high-performance hydrogen storage systems.

In this work, we perform a comprehensive density functional theory (DFT) investigation to evaluate the hydrogen storage potential of pristine and metal-decorated C_8_ quantum dots (M–C_8_, M = Li, Mg, Ti). Our study aims to: (i) determine the most stable adsorption sites for Li, Mg, and Ti on the C_8_ framework and characterize the metal–support interaction; (ii) elucidate the mechanism of single H_2_ adsorption (physisorption vs. chemisorption) on pristine and M–C_8_ systems; (iii) quantify the sequential hydrogen uptake and the maximum gravimetric capacity for each decorated system; and (iv) employ grand canonical thermodynamic analysis to predict the reversible storage performance under practical temperature and pressure conditions. By combining electronic structure analysis, adsorption energetics, and thermodynamic modeling, this study provides fundamental insights into how metal functionalization tailors the hydrogen storage properties of ultrasmall carbon clusters, paving the way for their rational design as high-performance, lightweight storage materials.

## 2. Computational Methodology

All computations were carried out within the Kohn–Sham density functional theory (DFT) framework using the ωB97X-3c functional [29] together with the valence double-ζ polarized (vDZP) basis set, as implemented in the ORCA 6 quantum chemistry package [30]. The ωB97X-3c composite method, developed by Grimme and co-workers, employs a range-separated hybrid exchange–correlation functional in combination with a compact yet well-balanced basis set. It further incorporates the atom-pairwise D4 dispersion correction and the geometrical counterpoise (gCP) scheme to account for basis-set superposition error (BSSE), ensuring reliable energetics and optimized structures [29].

This methodological setup provides accurate geometries and reliable interaction energies for noncovalent and weakly chemisorptive systems at a relatively low computational cost, making it well suited for investigating hydrogen adsorption on C_8_ nanocage quantum dots. The pristine C_8_ nanocage, its metal-decorated derivatives (M-CQD; M = Li, Mg, and Ti), and all corresponding hydrogen-bound complexes were fully optimized without imposing symmetry constraints. Self-consistent field (SCF) convergence criteria were set to 10^−6^ a.u. for total energy and 10^−8^ a.u. for density, with a maximum gradient threshold of 3 × 10^−4^ a.u. Vibrational frequency calculations were performed at the same ωB97X-3c/vDZP level to verify that each optimized structure represents a true minimum on the potential energy surface (i.e., no imaginary frequencies) and to obtain zero-point energy (ZPE) and thermal corrections at 298 K and 1 atm. For the pristine C_8_ nanocage, all vibrational modes were real, confirming its structural stability, which is consistent with previous theoretical findings [18]. To further assess the intrinsic stability of both pristine and metal-decorated C_8_ nanocages, the cohesive energy (*E*_coh_) was also calculated. The cohesive energy per atom was determined using the standard expression [31]:

(1) $$E_{\mathrm{coh}}=\frac{E_{\mathrm{total}}-\sum_{i}n_{i}E_{i}}{N}$$

where *E*_total_ is the total electronic energy of the fully optimized C_8_ (pristine or decorated) system, *E*_*i*_ is the atomic energy of the isolated atom *i*, *n*_*i*_ is the number of atoms of type *i*, and *N* is the total number of atoms in the nanocage. A more negative *E*_coh_ indicates a structurally more stable configuration. Cohesive energies were evaluated at 0 K to assess the intrinsic structural stability of the C_8_-based systems, while temperature- and pressure-dependent effects relevant to hydrogen storage were treated separately through vibrational analysis and grand canonical thermodynamic modeling.

The binding energy of a single Li, Mg, or Ti atom on the carbon nanocage was calculated to assess the strength of the metal–adsorbent interaction. For a given metal atom *M* adsorbed on the nanocage, the binding energy was determined as:

(2) $$E_{b}=E_{\text{M-CQD}}-E_{\mathrm{CQD}}-E_{M}$$

where $E_{\text{M-CQD}}$ is the total energy of the optimized metal-decorated C_8_ nanocage, $E_{\mathrm{CQD}}$ is the total energy of the pristine C_8_ nanocage, and $E_{M}$ is the energy of the isolated Li, Mg or Ti atom in the gas phase. All energies were obtained at the same level of theory. Negative values of $E_{b\;}$ indicate exothermic and thermodynamically favorable binding of the metal atom to the nanocage. Because ωB97X-3c inherently includes both dispersion (D4) and gCP corrections, no additional empirical or BSSE corrections were applied, ensuring internal consistency of all calculated interaction energies.

Spin-state effects were examined for the metal-decorated C_8_ systems. The singlet state was identified as the lowest-energy configuration for Mg–C_8_, while the triplet state was found to be energetically preferred for Ti–C_8_ and was therefore used in all subsequent calculations. Neutral Li–C_8_ was treated in its open-shell doublet ground state. The calculated 〈S^2^〉 values are close to the ideal values, indicating negligible spin contamination (Table S1).

The adsorption energy for one hydrogen molecule adsorbed on a metal-decorated nanocage was calculated as:

(3) $$E_{\mathrm{ads}}=E_{H2/\text{M-CQD}}-E_{\text{M-CQD}}-E_{H2}$$

where *E*_H2/M-CQD_ is the total electronic energy of the optimized H_2_ adsorbed complex, *E*_M-CQD_ is the energy of the isolated metal-decorated nanocage, and *E*_H2_ is the energy of an isolated H_2_ molecule optimized at the same level of theory. Zero-point energy corrections associated with H_2_ vibrational modes were evaluated for representative adsorption configurations. The resulting ΔZPE contributions are small (≤0.02 eV per H_2_) and therefore were not explicitly included in the reported adsorption energies, as they do not affect the relative trends or the qualitative conclusions of this comparative study.

For systems containing multiple adsorbed H_2_ molecules, the average adsorption energy per H_2_ molecule was determined as:

(4) $${\bar{E}}_{\mathrm{ads}}=\frac{1}{n}\;\left[E_{\mathrm{nH}2/\text{M-CQD}}-E_{\text{M-CQD}}-nE_{H2\;}\right]$$

where *n* is the number of hydrogen molecules adsorbed on the metal-decorated nanocage.

This expression provides an effective measure of the average interaction strength during sequential hydrogen adsorption.

The interactions of hydrogen molecules with metal-decorated C_8_ clusters were investigated using the localized molecular orbital energy decomposition analysis (LMOEDA) method developed by Su and Li [32]. Calculations were performed with the GAMESS-US 2023 version R1 [33,34,35] using the MN15 functional [36]. A def2-TZVP basis set was employed for all atoms. This approach allows the total interaction energy to be partitioned into its fundamental contributions (electrostatic, exchange, polarization, repulsion, and dispersion), providing a detailed understanding of the physical forces governing hydrogen adsorption. Both single and multi-H_2_ adsorption configurations were examined.

The nature of intermolecular interactions and bonding characteristics within the investigated systems was examined through interaction region indicator (IRI) analysis [37,38], performed using the Multiwfn package (version 38). Three-dimensional IRI isosurfaces were subsequently visualized with the visual molecular dynamics (VMD) software version 1.9.3 [39], allowing a detailed spatial interpretation of the interaction features.

The gravimetric hydrogen storage capacity (wt%) was determined using the following equation [40]:

(5) $$\mathrm{wt}\%=\frac{nM_{H2}}{M_{\text{M-CQD}}+nM_{H2}}\times 100$$

where *M*_H2_ and *M*_M-CQD_ are the molar masses of hydrogen and metal-decorated CQD, respectively.

## 3. Results and Discussion

The structural stability, electronic properties, and hydrogen adsorption behavior of pristine and metal-decorated C_8_ quantum dots were analyzed using the composite method ωB97X-3c. The influence of Li, Mg, and Ti decoration on the geometry, electronic structure, and adsorption energetics of the C_8_ nanocage is systematically compared.

### 3.1. Structure and Stability of Pristine and Metal-Decorated CQD

The structural stability of pristine and metal-decorated C_8_ quantum dots is a key factor determining their potential applications in hydrogen storage. Metal adsorption can induce substantial modifications in bond lengths, bond angles, and overall cage symmetry, which in turn affect the electronic properties and adsorption behavior of the system. In this subsection, the optimized geometries, cohesive energies, and metal binding strengths of pristine C_8_ and M–C_8_ (M = Li, Mg, Ti) are analyzed to establish a clear correlation between structural modification and energetic stability.

**Figure 1:**
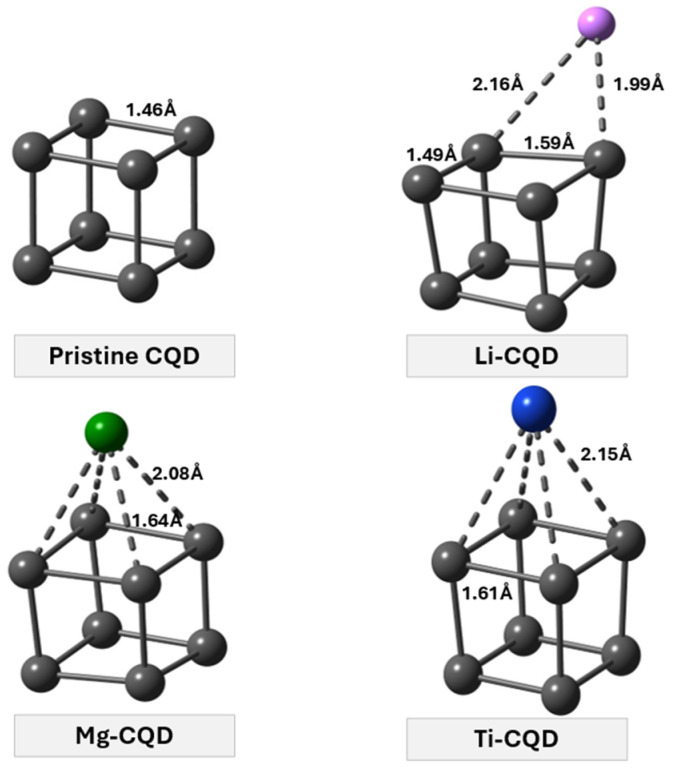
Optimized geometries of pristine and metal-decorated C_8_ quantum dots (M-CQD, M = Li, Mg, Ti).

Figure 1 shows the optimized geometries of the pristine C_8_ quantum dot and its metal-decorated derivatives (Li–C_8_, Mg–C_8_, and Ti–C_8_), while the corresponding structural, energetic, and electronic parameters are summarized in Table 1. The pristine C_8_ nanocage adopts a highly symmetric cage-like geometry with uniform C–C bond lengths of approximately 1.46 Å and C–C–C angles tightly distributed around 90° (89.99–90.01°). These values are in excellent agreement with previously reported first-principles studies on isolated C_8_ nanocages, which identified C_8_ as a structurally stable, weakly strained carbon cluster with high geometric symmetry. The large HOMO–LUMO gap of 7.440 eV further confirms the electronically inert nature of the isolated C_8_ quantum dot, in agreement with earlier theoretical reports.

Metal decoration induces clear but metal-dependent deviations from the structural characteristics of isolated C_8_. In the Li–C_8_ system, C–C bond lengths expand to 1.49–1.59 Å, and the C–C–C angles broaden significantly to 81.12–98.35°, indicating a substantial departure from the near-ideal geometry of pristine C_8_. This distortion reflects the sensitivity of the small C_8_ cage to external charge perturbation, as electron donation from Li weakens selected C–C interactions and increases the structural flexibility of the cage compared to the isolated nanocage. Despite this deformation, the cohesive energy remains highly negative (−4.673 eV), demonstrating that the Li-decorated structure retains thermodynamic stability.

For Mg–C_8_, the deviation from the isolated C_8_ geometry is more moderate. The average C–C bond length increases to about 1.643 Å, while the angular distribution (83.38–97.17°) remains narrower than that observed for Li–C_8_. Compared to isolated C_8_, this behavior suggests a balanced interaction in which the carbon framework is perturbed but not excessively distorted. The cohesive energy of −4.459 eV confirms that Mg decoration preserves the overall stability of the nanocage, albeit with a slightly reduced rigidity relative to pristine C_8_.

Ti–C_8_ exhibits a distinct stabilization pattern compared to isolated C_8_. Although Ti binds strongly to the carbon cage, the resulting structural distortion is limited, with C–C–C angles confined to 86.76–93.30°, close to those of the pristine nanocage. The cohesive energy of Ti–C_8_ (−4.923 eV) is even more negative than that of isolated C_8_ (−4.881 eV), indicating enhanced structural stabilization upon Ti decoration. This suggests that Ti incorporation compensates for the geometric perturbation through strong metal–carbon interactions, thereby maintaining the integrity of the C_8_ framework.

Metal decoration also leads to systematic changes in the electronic structure relative to isolated C_8_. While pristine C_8_ displays a wide HOMO–LUMO gap of 7.440 eV, Li decoration dramatically reduces the gap to 1.715 eV due to a pronounced upward shift in the HOMO level, signaling strong electronic activation compared to the isolated nanocage. In contrast, Mg and Ti decoration result in more moderate gap reductions (6.824 and 6.136 eV, respectively), indicating controlled electronic tuning while largely preserving the electronic character of pristine C_8_.

Overall, relative to the isolated C_8_ quantum dot reported in the literature, metal decoration provides an effective strategy to tailor both structural flexibility and electronic reactivity. While the pristine C_8_ nanocage is structurally rigid and electronically inert, Li, Mg, and Ti decoration progressively introduce geometric distortion and electronic activation to different extents, establishing a clear structure–property relationship that underpins the hydrogen adsorption behavior discussed in the following sections.

### 3.2. Single H_2_ Adsorption Mechanism

**Figure 2:**
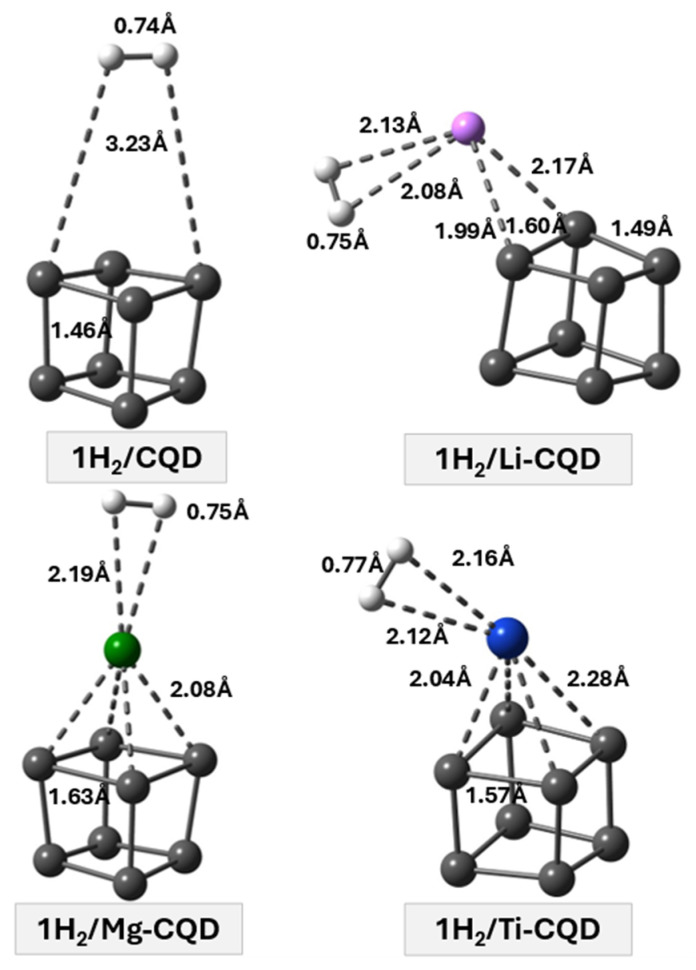
Optimized adsorption configurations of a single H_2_ molecule on pristine and metal-decorated C_8_ quantum dots (M-CQD, M = Li, Mg, Ti).

Several initial adsorption configurations were examined for a single H_2_ molecule on pristine and metal-decorated C_8_ quantum dots, including different adsorption sites and molecular orientations. For each system, full structural optimizations were performed, and the most stable adsorption geometry was identified based on the lowest total energy. The optimized configurations corresponding to the most stable adsorption states are shown in Figure 2. For pristine C_8_, the H_2_ molecule is adsorbed at a relatively large distance from the carbon cage (≈3.23 Å), with a low adsorption energy of −0.062 eV. The H–H bond length remains essentially unchanged (0.74 Å), indicating that the molecular integrity of H_2_ is preserved upon adsorption. Notably, this adsorption energy lies outside the commonly accepted optimal range for reversible hydrogen storage (−0.100 to −0.600 eV per H_2_ molecule). Consequently, pristine C_8_ is not suitable as an efficient hydrogen storage material under practical conditions. This limitation provides a clear rationale for employing metal decoration to enhance the interaction strength and achieve adsorption energies within the desirable range.

Metal decoration significantly strengthens the interaction between H_2_ and the C_8_ quantum dot. In the Li-CQD system, the adsorption energy increases to −0.172 eV, and the optimized geometry shows that H_2_ is preferentially located near the Li atom, with Li–H distances in the range of 1.99–2.08 Å. The H–H bond length exhibits a slight elongation to approximately 0.75 Å, while the H_2_ molecule remains intact.

These structural characteristics, combined with the moderate adsorption energy, confirm that hydrogen adsorption occurs through a physisorption mechanism, representing a noticeable improvement in interaction strength compared to pristine C_8_.

A further increase in adsorption strength is observed for Mg-CQD, where the adsorption energy reaches −0.304 eV. The H_2_ molecule is positioned closer to the Mg atom, with Mg–H distances of about 2.08–2.19 Å, and the H–H bond length remains close to 0.75 Å. Compared to Li-CQD, the shorter adsorption distances and larger adsorption energy reflect a stronger interaction between H_2_ and the Mg-decorated system.

The strongest single H_2_ adsorption is obtained for Ti-CQD, with an adsorption energy of −0.451 eV. In this case, the H–H bond length increases to approximately 0.77 Å, indicating a more pronounced elongation compared to the isolated H_2_ molecule. The optimized geometry shows that H_2_ is located in close proximity to the Ti atom, with Ti–H distances of about 2.04–2.16 Å, while remaining in molecular form.

**Figure 3:**
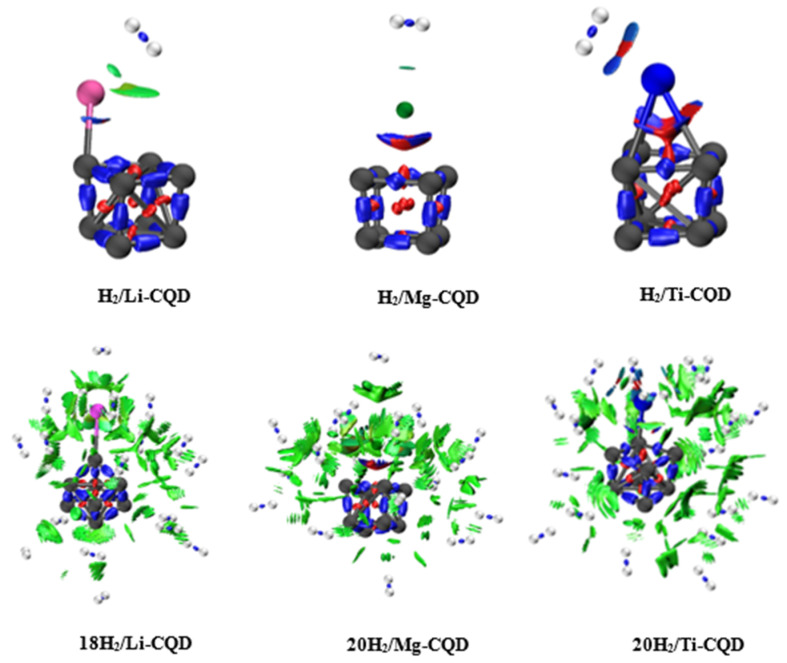
IRI isosurface plots (Isovalue = 0.7 a.u.) of the systems H_2_/Li-CQD, H_2_/Mg-CQD, H_2_/Ti-CQD, 18H_2_/Li-CQD, 20H_2_/Mg-CQD, and 20H_2_/Ti-CQD.

To further elucidate the physical origin of the H_2_ interaction and rationalize the observed adsorption trends, the interaction between a single H_2_ molecule and the metal-decorated C_8_ quantum dots was analyzed using localized molecular orbital energy decomposition analysis LMOEDA (Table 2) and interaction region indicator IRI analysis (Figure 3). The energetic decomposition reveals clear metal-dependent interaction mechanisms governing hydrogen adsorption.

For the Li-CQD system, the interaction with H_2_ is dominated by weak dispersion forces (−0.343 eV), accompanied by modest electrostatic (−0.118 eV) and polarization (−0.067 eV) contributions, which are partially counterbalanced by Pauli repulsion (0.353 eV). As a result, the total interaction energy remains small (−0.151 eV), confirming that hydrogen adsorption on Li-CQD is governed by weak physisorption. This interpretation is fully consistent with the relatively large Li–H distances, minimal H–H bond elongation, and moderate adsorption energy obtained from DFT calculations.

In the case of Mg-CQD, both electrostatic (−0.217 eV) and polarization (−0.219 eV) contributions are significantly enhanced compared to Li-CQD, reflecting stronger charge-induced interactions between the Mg atom and the H_2_ molecule. Although the repulsive component increases to 0.697 eV, the overall interaction remains stabilizing (−0.227 eV), indicating a more balanced adsorption mechanism. This energetic profile explains the intermediate adsorption strength of Mg-CQD, which lies within the optimal window for reversible hydrogen storage while preserving molecular H_2_ adsorption.

The Ti-CQD system exhibits the strongest interaction with a single H_2_ molecule. The energetic decomposition shows pronounced electrostatic (−0.396 eV) and polarization (−0.359 eV) contributions, together with the largest dispersion component (−0.757 eV). Despite the higher repulsion term (1.020 eV), these attractive interactions dominate, leading to a substantially more stabilizing total interaction energy of −0.477 eV. This strong interaction correlates with the shorter Ti–H distances and the more pronounced elongation of the H–H bond, indicating enhanced electronic coupling between Ti and H_2_.

These energetic trends are further supported by IRI analysis. For Li-CQD and Mg-CQD, the interaction regions between the metal atom and the H_2_ molecule are characterized by diffuse green isosurfaces, indicative of weak dispersive and van der Waals interactions. The absence of blue bonding regions confirms that hydrogen remains molecularly adsorbed through physisorption. In contrast, the Ti-CQD system shows a transition from green to bluish-cyan isosurfaces in the Ti–H_2_ interaction region, reflecting a strengthening of the interaction toward a more polarized or partially covalent character.

Overall, the combined energetic decomposition and IRI analyses provide a consistent physical picture of single H_2_ adsorption on metal-decorated C_8_ quantum dots, revealing a progressive strengthening of the interaction from Li-CQD to Mg-CQD and Ti-CQD. These findings rationalize the observed adsorption energies and establish a clear link between electronic interaction mechanisms and hydrogen binding strength, forming a solid basis for understanding the subsequent sequential hydrogen adsorption behavior.

To rigorously validate the computational methodology, both basis-set and functional effects were systematically examined by jointly analyzing adsorption energies and optimized geometries. First, to assess basis-set sensitivity, adsorption complexes were recalculated using the same underlying ωB97X functional [41] while increasing the basis from the compact vDZP employed in the composite ωB97X-3c scheme to the larger def2-QZVP basis [42]. The resulting adsorption energies exhibit an excellent linear correlation (Figure S1; y = 1.087x − 0.010, R^2^ = 0.990), with small deviations of +0.022, +0.029, +0.009, and +0.064 eV for H_2_/CQD, H_2_/Li-CQD, H_2_/Mg-CQD, and H_2_/Ti-CQD, respectively (MAE = 0.031 eV; RMSE = 0.037 eV). Importantly, the optimized geometries remain equally robust upon basis enlargement: the CQD framework is essentially unchanged, with average C–C distances varying only from 1.46 to 1.48 Å (Table S2), while metal–carbon anchoring distances differ by ≤ 0.02 Å (e.g., Li–C ≈ 2.21–2.22 Å; Mg–C ≈ 2.42–2.43 Å; Ti–C ≈ 2.21–2.23 Å). Adsorption contact distances show only modest variations consistent with the small energetic differences (e.g., H_2_⋯C: 2.96 Å vs. 2.94 Å; H_2_⋯Li: ~0.03 Å difference; H_2_⋯Mg: ~0.04 Å difference), confirming structural reproducibility.

Second, functional dependence was evaluated at a fixed def2-TZVP basis [42] using ωB97X, ωB97X-V [43], ωB97M-V [44], and dispersion-corrected PBE-D4 [45], M06-2X-D4 [46], and B3LYP-D4 [47]. While absolute adsorption energies vary across exchange–correlation functionals, the numerical differences remain within an expected and physically reasonable range for weak H_2_ physisorption. For instance, at the def2-TZVP level, the adsorption energy of H_2_ on Ti-CQD varies from −0.321 eV (PBE-D4) to −0.506 eV (ωB97X), with ωB97X-V and M06-2X-D4 predicting intermediate values (−0.492 and −0.391 eV, respectively) (Table S2). For Mg-CQD, the corresponding range is narrower, from −0.181 eV (PBE-D4) to −0.304 eV (ωB97X), while for Li-CQD, adsorption energies vary between −0.110 eV (PBE-D4) and −0.195 eV (B3LYP-D4). In the case of pristine CQD, the spread remains modest, from −0.010 eV (PBE-D4) to −0.079 eV (ωB97X). Importantly, despite these quantitative variations, the adsorption-strength ordering Ti-CQD > Mg-CQD > Li-CQD > CQD is preserved across all dispersion-corrected functionals (Figure S2). This consistency is statistically confirmed by a Spearman rank correlation coefficient of *ρ* = 1.00 for ωB97X, ωB97X-V, PBE-D4, and M06-2X-D4, indicating full preservation of energetic trends. Even for B3LYP-D4, where Li-CQD and Mg-CQD differ by only ~0.003 eV, the deviation is negligible relative to the characteristic energy scale of physisorption. The geometric analysis further reinforces this robustness. Across functionals, the CQD framework remains essentially invariant, with average C–C distances confined to 1.46–1.48 Å (Table S2). Metal–carbon anchoring distances show only minimal variation: Li–C = 2.21–2.22 Å, Mg–C = 2.42–2.43 Å, and Ti–C = 2.21–2.23 Å. Adsorption contact distances follow the energetic trends in a physically consistent manner. For example, the shortest H_2_⋯Ti interaction distance ranges from ~2.02 Å (ωB97X) to ~2.17 Å (PBE-D4), correlating with the stronger adsorption predicted by range-separated hybrids. Similarly, H_2_⋯Mg distances vary modestly from 2.47 Å to 2.51 Å, while H_2_⋯Li contacts remain around 2.82–2.85 Å, consistent with their weaker binding energies. In all cases, the H–H bond length remains essentially unchanged (≈0.74 Å), confirming purely molecular adsorption.

Collectively, these quantitative energetic and geometric benchmarks demonstrate that both the magnitude of adsorption and the inferred cooperative physisorption mechanism are insensitive to functional choice within dispersion-corrected density functional theory. The consistency of energy ordering, contact distances, and framework stability provides strong evidence that the mechanistic interpretation is robust and reproducible, thereby validating the reliability of the ωB97X-3c description adopted in this work.

### 3.3. Sequential H_2_ Adsorption and Maximum Capacity

The sequential adsorption of multiple H_2_ molecules on metal-decorated C_8_ quantum dots was investigated to assess the evolution of adsorption strength as a function of hydrogen coverage and to determine the maximum gravimetric storage capacity of each system. For Li-CQD, Mg-CQD, and Ti-CQD, H_2_ molecules were incrementally added, with the most energetically favorable configuration at each stage identified through full structural optimization.

Across all systems, the first H_2_ molecule exhibits the most negative average adsorption energy, reflecting the availability of the most favorable adsorption sites. As hydrogen coverage increases, the average adsorption energy per molecule (${\overset{ˉ}{E}}_{\mathrm{ads}}$) becomes progressively less negative, indicating a gradual weakening of the interaction due to steric crowding and the finite number of energetically optimal binding sites.

In the Ti-CQD system, the initial H_2_ adsorption induces a notable elongation of the H–H bond to 0.771 Å, with a strong average adsorption energy of −0.451 eV (Table 3). Subsequent adsorption results in a slight shortening of the H–H bond to 0.747–0.748 Å, while (${\overset{ˉ}{E}}_{\mathrm{ads}}$) remains within the optimal range for reversible storage (−0.100 to −0.600 eV per H_2_ molecule) up to *n* = 18. Beyond this coverage, the adsorption energy approaches the lower bound of the optimal range, indicating that *n* = 18 represents the practical maximum hydrogen uptake for Ti-CQD. Despite the strong binding at low coverage, the relatively high atomic mass of Ti limits the gravimetric storage capacity to 21.75 wt%.

For Mg-CQD, the initial adsorption energy is moderate (−0.304 eV) yet well within the optimal range. As H_2_ coverage increases, (${\overset{ˉ}{E}}_{\mathrm{ads}}$) decreases smoothly, approaching −0.100 eV at *n* ≈ 18–20, while the H–H bond length remains nearly constant (≈0.747–0.750 Å), confirming molecular physisorption throughout the adsorption sequence. The combination of substantial hydrogen uptake and the moderate atomic mass of Mg yield a high theoretical gravimetric storage capacity of 24.95 wt%.

Li-CQD exhibits comparatively weaker interactions with H_2_. The average adsorption energy decreases more rapidly with increasing coverage, approaching the lower limit of the optimal range at *n* ≈ 18. Consequently, Li-CQD demonstrates a lower maximum hydrogen uptake relative to Mg- and Ti-CQDs. Nevertheless, due to the very low atomic mass of Li, this system achieves the highest theoretical gravimetric capacity among the studied materials, reaching 25.91 wt%.

The sequential adsorption procedure inherently captures adsorbate–adsorbate interactions and steric effects. At each step, all structures were fully optimized, allowing hydrogen molecules to reorganize in response to mutual repulsion, steric crowding, and competition for favorable interaction sites. These effects manifest as a gradual reduction in (${\overset{ˉ}{E}}_{\mathrm{ads}}$), reflecting the progressive saturation of optimal adsorption environments. The emergence of weaker binding at high coverage therefore defines the practical maximum hydrogen uptake, preventing overestimation of storage capacity. LMOEDA and IRI analyses further confirm that these interactions are cooperative yet competitive, with increasing dispersion-dominated stabilization accompanied by enhanced repulsive contributions at high loading.

To elucidate the underlying adsorption mechanisms at high coverage, multi-H_2_ adsorption complexes were analyzed using LMOEDA and IRI approaches. For the 18H_2_/Li-CQD system, energy decomposition analysis reveals that dispersion interactions (−3.046 eV) dominate stabilization, while electrostatic (−0.847 eV) and polarization (−0.372 eV) contributions are comparatively modest. These attractive interactions are partially offset by Pauli repulsion (2.535 eV) and a positive exchange term (0.435 eV), yielding a moderate total interaction energy of −1.296 eV. This profile demonstrates that hydrogen uptake in Li-CQD at high coverage is primarily governed by collective van der Waals forces rather than directional bonding, explaining the progressive weakening of (${\overset{ˉ}{E}}_{\mathrm{ads}}$) with increasing hydrogen loading.

In the 20H_2_/Mg-CQD system, both electrostatic (−1.582 eV) and polarization (−1.349 eV) contributions increase substantially compared to Li-CQD, in addition to a strong dispersion component (−4.410 eV). Although the repulsive term rises to 5.415 eV, the overall interaction remains stabilizing (−1.889 eV). This balance between attractive and repulsive contributions indicates a cooperative adsorption mechanism, in which multiple H_2_ molecules are stabilized by the combined effects of dispersion forces and charge-induced interactions. Such a balance accounts for the sustained adsorption energies within the optimal reversible storage window and the high hydrogen uptake observed for Mg-CQD.

For the 20H_2_/Ti-CQD complex, the energetic decomposition shows the strongest overall stabilization among the studied systems. Pronounced electrostatic (−2.447 eV) and polarization (−3.610 eV) contributions, together with the largest dispersion term (−4.848 eV), dominate the interaction, effectively compensating for the substantial Pauli repulsion (9.181 eV). Despite this strong stabilization, excessively strong interactions at high hydrogen coverage can lead to increased desorption losses under practical operating conditions, as indicated by the thermodynamic analysis.

The IRI analysis provides further support for these energetic trends. For all multi-H_2_ adsorption systems, the interaction regions surrounding the adsorbed hydrogen molecules are characterized by extended and diffuse green isosurfaces, indicating that weak noncovalent interactions dominate at high coverage. The lack of pronounced blue bonding regions confirms that hydrogen remains molecularly adsorbed, even at maximum loading. Moreover, the lack of significant red regions suggests minimal steric repulsion within the adsorption region, allowing multiple H_2_ molecules to be accommodated without structural destabilization of the C_8_ framework.

Compared to single H_2_ adsorption, the more spatially extended green IRI features observed for multi-H_2_ systems reflect a cooperative adsorption mechanism, in which collective dispersive and electrostatic interactions stabilize the hydrogen ensemble. Such cooperative physisorption is especially beneficial for hydrogen storage applications, as it allows high gravimetric capacities to be achieved while maintaining favorable adsorption–desorption kinetics and reversibility. The combined energetic decomposition and IRI analyses therefore provide a consistent physical explanation for the superior reversible hydrogen storage performance of Mg-CQD relative to Li- and Ti-decorated systems.

### 3.4. Thermodynamic and Kinetic Analysis of Reversible Storage

To assess the practical feasibility of hydrogen storage on metal-decorated C_8_ quantum dots, a combined thermodynamic and kinetic analysis was carried out under representative temperature and pressure conditions. The calculated desorption temperatures (*T_D_*), desorption energies (*E*_des_), residence times (*τ*), and reversible storage metrics are summarized in Table 4 for Li-CQD, Mg-CQD, and Ti-CQD systems.

The desorption temperature provides a direct measure of the thermal stability of adsorbed hydrogen using the Van’t Hoff equation [48]:

(6) $$T_{D}=-\frac{E_{\mathrm{ads}}}{k_{B}(\frac{\Delta S}{R}-lnP)}$$

*T_D_* incorporates variables such as adsorption energy (*E*_ads_), the equilibrium pressure (*P*), and the entropy change of hydrogen molecules transitioning from gaseous phase to liquid phase. Additionally, *k_B_* and *R* represent the Boltzmann constant and the ideal gas constant, respectively. The entropy change Δ*S* was taken as 75.44 J·mol^−1^·K^−1^ [49,50], corresponding to the dominant entropy loss associated with the transition of molecular hydrogen from the gas phase to an adsorbed state. Since hydrogen adsorption remains molecular and physisorptive in all investigated systems, Δ*S* is largely governed by gas-phase contributions and is weakly dependent on the specific adsorbent, while system-specific effects are reflected in the adsorption energies.

At 1 bar, *T_D_* increases from 76.71 K for Li-CQD to 102.28 K for Mg-CQD and 127.85 K for Ti-CQD, reflecting progressively stronger hydrogen binding. With increasing pressure, *T_D_* rises systematically for all systems, reaching 155.76 K (Li-CQD), 207.68 K (Mg-CQD), and 259.61 K (Ti-CQD) at 100 bar. This pressure dependence indicates that hydrogen release can be effectively regulated by operating conditions, which is essential for reversible storage.

Importantly, the calculated desorption energies (*E*_des_) are consistently lower in magnitude than the corresponding average adsorption energies (*Ē*_ads_) for all metal-decorated C_8_ quantum dots. This energetic hierarchy ensures that hydrogen release requires less energy than hydrogen uptake, thereby confirming the thermodynamic reversibility of the storage process and enabling hydrogen desorption under moderate thermal conditions.

The kinetic stability of the adsorbed hydrogen was evaluated through the residence times *τ*. The desorption time can be estimated using the following equation [51]:

(7) $$\tau =\frac{exp(-E_{\mathrm{ads}}/k_{B}T)}{\nu_{0}}$$

where *v*_0_ is the attempt frequency (10^12^ s^−1^).

At 233 K, *τ* increases from 0.03 ns for Li-CQD to 0.06 ns for Mg-CQD and 0.15 ns for Ti-CQD, consistent with the increasing interaction strength. At 298 K, *τ* decreases to values between 0.02 and 0.10 ns for all systems, indicating that hydrogen desorption remains kinetically accessible and does not suffer from trapping effects.

Although adsorption energies, desorption temperatures, and kinetic release times describe the intrinsic thermodynamic behavior of H_2_/M-CQD interactions, they do not provide a direct measure of the reversibly accessible hydrogen capacity under practical operating conditions. To evaluate the operational storage performance, we calculate the grand canonical partition function (*z*), the hydrogen chemical potential *μ*(*P*,*T*), and the resulting average number of adsorbed molecules N_avg_ (*P*,*T*), which together determine the reversible uptake N_A_, the desorbed fraction N_D_, and ultimately the usable storage N_P_ = N_A_ − N_D_ and C_E_ (wt%). In this framework, the adsorption probability of each configuration enters through the grand canonical partition function (*z*). The equation to calculate (*z*) is defined as follows [21]:

(8) $$z=1+\sum_{i=1}^{n}exp(\frac{E_{i,\mathrm{ads}}-\mu (P,T)}{k_{B}T})$$

where *n* is the maximum number of adsorbed H_2_ molecules, $E_{i,\mathrm{ads}}$ is the adsorption energy of the *i*th H_2_ molecule, $k_{B}$ is the Boltzmann constant (1.38 × 10^−23^ J K^−1^), and μ(P,T) is the chemical potential of molecular hydrogen in the gas phase at a given pressure (*P*) and temperature (*T*).

The chemical potential μ(P,T) can be determined from thermodynamic relations or from experimental data, and it is given by:

(9) $$\mu_{H_{2}}(P,T)=\Delta H+T\Delta S+k_{B}Tln\frac{P}{P_{0}}$$

where ΔH, ΔS, and $P_{0}\;$ represent the enthalpy change, entropy change, and standard atmospheric pressure (1.01 × 10^5^ Pa), respectively. In this expression, the term ΔH+TΔS describes the free energy contribution of the hydrogen gas phase, while the logarithmic term accounts for the dependence on external pressure. The values of ΔH and ΔS  were taken from the experimental database [22], which provides reliable thermodynamic parameters for H_2_ adsorption and desorption on metal-decorated systems. In the grand canonical framework, the chemical potential, *μ*(*P*,*T*), defines the thermodynamic state of the hydrogen gas phase and is independent of the adsorbent. System-specific effects, including curvature, quantum confinement, and metal-induced charge transfer, are fully incorporated through the adsorption energies within the partition function, which in turn determine hydrogen occupancy and the reversible storage capacity.

The average number of adsorbed hydrogen molecules at a given pressure and temperature, $N_{\mathrm{avg}}(P,T)$, is derived from the partition function and is expressed as:

(10) $$N_{\mathrm{avg}}(P,T)=N_{T}[\frac{z-1}{z}]$$

**Figure 4:**
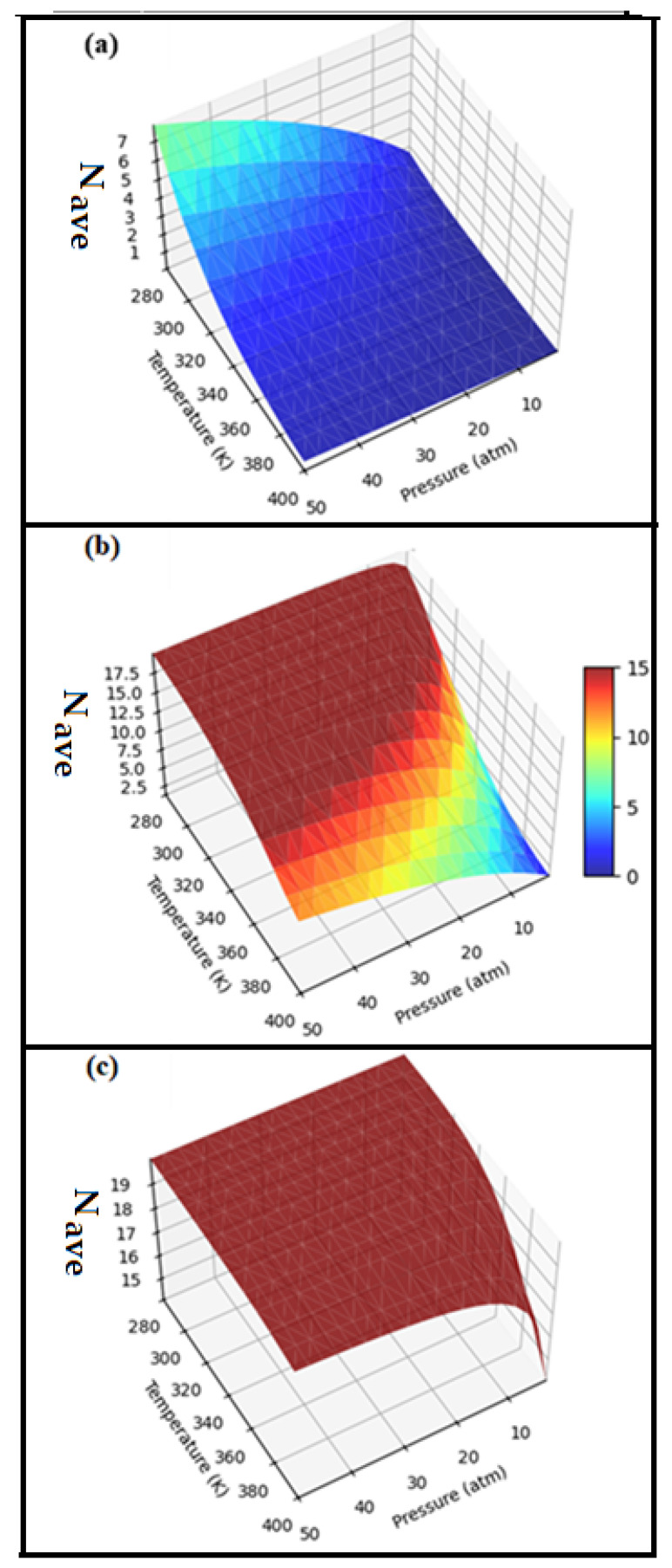
Average number of H_2_ molecules (*N*_ave_) adsorbed on (**a**) Li-CQD, (**b**) Mg-CQD, and (**c**) Ti-CQD as a function of practical temperatures and pressures.

Figure 4 illustrates the variation in the average number of adsorbed H_2_ molecules (*N*ₐᵥₑ) as a function of temperature and pressure under practical operating conditions. For all systems, *N*ₐᵥₑ increases with increasing pressure and decreases with increasing temperature, reflecting the thermodynamic balance between hydrogen adsorption and desorption. Among the studied materials, Mg-CQD maintains a high *N*ₐᵥₑ over a broad range of temperatures and pressures, indicating a favorable compromise between hydrogen uptake and release. In contrast, Li-CQD exhibits lower *N*ₐᵥₑ values due to weaker hydrogen binding, while Ti-CQD shows a pronounced sensitivity to temperature, leading to reduced average hydrogen loading under practical conditions.

The reversible storage performance was further quantified using the thermodynamic descriptors N_T_, N_A_, N_D_, and N_P_ reported in Table 3. While Mg-CQD and Ti-CQD both exhibit high total hydrogen uptake (N_T_ = 20), their practical behavior differs markedly. Mg-CQD combines a high number of adsorbed hydrogen molecules (N_A_ = 19.41) with a relatively low number of desorbed species (N_D_ = 2.75), resulting in a large pool of reversibly stored hydrogen (N_P_ = 16.66). In contrast, Ti-CQD shows a high N_D_ value (17.66), which significantly limits the amount of practically usable hydrogen (N_P_ = 2.34). Li-CQD displays limited hydrogen uptake and therefore a lower reversible storage fraction. Although Ti-CQD exhibits strong H_2_ binding at low coverage, the excessive interaction strength leads to high desorption losses under practical conditions, severely limiting its reversible storage efficiency.

These trends are directly reflected in the effective reversible gravimetric capacities (C_E_). Among the studied systems, Mg-CQD achieves the highest reversible hydrogen storage capacity (21.7 wt%), significantly outperforming Li-CQD (6.16 wt%) and Ti-CQD (3.1 wt%). The strong initial Ti–H_2_ interaction leads to excessive sensitivity to temperature, causing rapid hydrogen loss under practical conditions. This superior performance arises from the balanced adsorption strength and moderate desorption energetics of Mg-CQD, which enable efficient hydrogen uptake while ensuring facile release under practical temperature and pressure conditions.

To further evaluate the relevance of the present results, the hydrogen storage performance of metal-decorated C_8_ quantum dots was compared with representative carbon-based hydrogen storage systems reported in the literature. A wide range of carbon nanostructures, including graphene, carbon nanotubes, fullerenes, and defect-engineered two-dimensional carbon frameworks, have been extensively investigated, often relying on metal decoration to enhance H_2_ adsorption strength (see Table 5).

The expanded comparison presented in Table 5 places the hydrogen storage performance of metal-decorated C_8_ quantum dots within a broader theoretical context, encompassing carbon-based nanostructures, metal–organic frameworks (MOFs), and covalent organic frameworks (COFs). Conventional carbon materials such as graphene, carbon nanotubes, and fullerenes typically exhibit moderate hydrogen adsorption energies (≈−0.15 to −0.35 eV per H_2_), leading to reversible gravimetric capacities generally below 10 wt% under practical conditions. Structural modifications through defects, heteroatom incorporation, or alternative carbon allotropes (e.g., biphenylene, irida-graphene, graphyne-based systems) can significantly enhance uptake, in some cases exceeding 10 wt% through optimized metal decoration.

Porous frameworks such as MOFs and COFs provide an alternative strategy by maximizing accessible surface area and hosting open metal sites. As shown in Table 5, selected MOFs (e.g., IRMOF-16, MOF-519, IRMOF-10) and metal-functionalized COFs can theoretically reach gravimetric capacities in the range of ~5–12 wt%, depending on pore architecture, metal species, and adsorption strength. However, these systems often rely on extended porous networks with relatively high framework mass, which can limit gravimetric efficiency despite favorable adsorption energetics.

In contrast, the ultrasmall size, extreme curvature, and full accessibility of adsorption sites in metal-decorated C_8_ quantum dots lead to a fundamentally different storage mechanism. The Mg-CQD system, in particular, combines an optimal adsorption energy (−0.30 eV per H_2_) with a lightweight carbon scaffold, resulting in a high predicted reversible gravimetric capacity of 21.7 wt%. This capacity surpasses most theoretical predictions reported for extended carbon nanostructures, MOFs, and COFs, while maintaining adsorption energies within the optimal window for reversible hydrogen storage. These results highlight the potential of molecular-scale, quantum-confined carbon systems as a complementary design paradigm to extended porous frameworks.

While the grand canonical analysis predicts a high reversible capacity for Mg–C_8_, it should be emphasized that this value represents an idealized theoretical upper limit based on isolated, well-dispersed metal decoration. Experimental realization may be limited by several factors, including (i) metal atom aggregation and surface diffusion, which reduce the density of active adsorption sites; (ii) difficulties in controlling single-atom loading and uniform coverage; (iii) possible structural reconstruction, defect formation, or contamination that can modify binding energetics; and (iv) kinetic limitations and stability under repeated adsorption–desorption cycling. Overcoming these challenges would likely require stabilization strategies such as defect/heteroatom anchoring, confinement within porous or hybrid matrices, or controlled synthesis routes combined with advanced characterization to verify metal dispersion and durability.

## 4. Conclusions

In the current work, a comprehensive density functional theory study was conducted to evaluate the hydrogen storage potential of pristine and metal-decorated C_8_ quantum dots. The influence of Li, Mg, and Ti decoration on structural stability, hydrogen adsorption mechanisms, and reversible storage performance was systematically investigated. The pristine C_8_ quantum dot was found to be structurally stable but unsuitable for hydrogen storage due to its very weak interaction with H_2_ (−0.06 eV), which lies outside the optimal energy window required for reversible adsorption. Metal decoration effectively activates the C_8_ nanocage, enabling adsorption energies within the desirable range while preserving molecular hydrogen adsorption. Single H_2_ adsorption energies of −0.17 eV, −0.30 eV, and −0.45 eV were obtained for Li-CQD, Mg-CQD, and Ti-CQD, respectively, accompanied by moderate H–H bond elongation. Sequential adsorption analysis revealed that Li-CQD can accommodate up to 18 H_2_ molecules, whereas Mg-CQD and Ti-CQD can host up to 20 H_2_ molecules, resulting in high theoretical gravimetric hydrogen storage capacities of 25.91 wt%, 24.95 wt%, and 21.75 wt%, respectively. Energetic decomposition and interaction region indicator analyses demonstrated that hydrogen uptake in all systems proceeds through a cooperative physisorption mechanism dominated by dispersion, electrostatic, and polarization interactions, with increasing metal-dependent contributions governing adsorption strength at higher hydrogen coverage. Thermodynamic and kinetic analyses further clarified the practical storage behavior. Although Ti-CQD exhibits strong hydrogen binding, excessive interaction strength leads to significant desorption losses under realistic operating conditions, limiting its reversible gravimetric capacity to 3.1 wt%. Li-CQD shows weaker hydrogen uptake, resulting in a moderate reversible capacity of 6.16 wt%. In contrast, Mg-CQD provides an optimal balance between adsorption stability and desorption accessibility, achieving a high reversible hydrogen storage capacity of 21.7 wt% over a broad range of temperatures and pressures.

It should be noted that the present study is based on an idealized theoretical model in which a single metal atom is assumed to be stably anchored to the C_8_ quantum dot. In practical experimental synthesis, metal aggregation, surface diffusion, or clustering may occur and represent a well-known challenge for metal-decorated nanostructures. Bridging this synthesis gap will require appropriate stabilization strategies, such as defect or heteroatom anchoring, confinement effects, or hybrid material design, which are beyond the scope of the present theoretical investigation.

Overall, Mg-decorated C_8_ quantum dots emerge as the most promising candidate among the studied systems for practical reversible hydrogen storage. The present results highlight the potential of ultrasmall carbon-based nanostructures as tunable and lightweight hydrogen storage materials and provide quantitative design guidelines for the development of next-generation nanostructured hydrogen storage systems.

## Figures and Tables

**Table 1:** Structural, energetic, and electronic parameters of pristine and metal-decorated C_8_ quantum dots using the wB97x-3c/vDZP level of theory.

Parameter	Pristine CQD	Li-CQD	Mg-CQD	Ti-CQD
C–C (Å)	1.46	1.49–1.59	1.64	1.61
C–C–C (Å)	89.99–90.01	81.12–98.35	83.38–97.17	86.76–93.30
M–C (Å)	-	1.99–2.16	2.08	2.15
E_coh_ (eV)	−4.881	−4.673	−4.459	−4.923
E_b_ (eV)	-	−2.956	−2.146	−5.250
E_LUMO_ (eV)	−2.903	−2.611	−1.946	−0.677
E_HOMO_ (eV)	−10.343	−4.326	−8.770	−6.813
∆E_g_ (eV)	7.440	1.715	6.824	6.136

**Table 2:** Energetic components governing single and multi-H_2_ adsorption on Li-, Mg-, and Ti-CQD systems.

E_component_ (eV)	H_2_/Li-CQD	H_2_/Mg-CQD	H_2_/Ti-CQD	18H_2_/Li-CQD	20H_2_/Mg-CQD	20H_2_/Ti-CQD
Electrostatic	−0.118	−0.217	−0.396	−0.847	−1.582	−2.447
Exchange	0.024	−0.137	0.015	0.435	0.038	−0.931
Polarization	−0.067	−0.219	−0.359	−0.372	−1.349	−3.610
Dispersion	−0.343	−0.351	−0.757	−3.046	−4.410	−4.848
Repulsion	0.353	0.697	1.020	2.535	5.415	9.181
Total energy	−0.151	−0.227	−0.477	−1.296	−1.889	−2.655

**Table 3:** Structural parameters and average adsorption and desorption energies for sequential H_2_ adsorption on metal-decorated C_8_ quantum dots (M-CQD, M = Li, Mg, Ti).

	Li-CQD			Mg-CQD			Ti-CQD		
n (H_2_)	${\overset{ˉ}{E}}_{\mathrm{ads}}$ (eV/H_2_)	*E*_des_ (eV)	H–H (Å)	${\overset{ˉ}{E}}_{\mathrm{ads}}$ (eV/H_2_)	*E*_des_ (eV)	H–H (Å)	${\overset{ˉ}{E}}_{\mathrm{ads}}$ (eV/H_2_)	*E*_des_ (eV)	H–H (Å)
1	−0.172	-	0.749	−0.304	-	0.752	−0.451	-	0.771
2	−0.167	−0.164	0.749	−0.295	−0.291	0.758	−0.393	−0.451	0.764
3	−0.155	−0.131	0.749	−0.269	−0.218	0.755	−0.362	−0.334	0.762
4	−0.141	−0.1	0.748	−0.232	−0.121	0.753	−0.3	−0.3	0.758
5	−0.123	−0.047	0.747	−0.194	−0.043	0.751	−0.225	−0.115	0.755
6	−0.11	−0.05	0.747	−0.171	−0.053	0.751	−0.22	0.077	0.753
7	−0.102	−0.053	0.746	−0.154	−0.053	0.75	−0.196	−0.194	0.752
8	−0.098	−0.069	0.746	−0.141	−0.054	0.749	−0.178	−0.053	0.751
9	−0.09	−0.027	0.746	−0.132	−0.057	0.749	−0.161	−0.054	0.75
10	−0.085	−0.042	0.746	−0.124	−0.055	0.749	−0.152	−0.023	0.75
11	−0.081	−0.039	0.745	−0.118	−0.056	0.749	−0.142	−0.074	0.749
12	−0.077	−0.038	0.745	−0.113	−0.06	0.748	−0.133	−0.038	0.749
13	−0.074	−0.039	0.745	−0.109	−0.056	0.748	−0.126	−0.04	0.748
14	−0.072	−0.036	0.745	−0.104	−0.043	0.748	−0.12	−0.045	0.748
15	−0.07	−0.039	0.745	−0.099	−0.034	0.748	−0.117	−0.041	0.748
16	−0.068	−0.043	0.745	−0.096	−0.043	0.748	−0.111	−0.069	0.748
17	−0.065	−0.026	0.745	−0.092	−0.037	0.748	−0.107	−0.018	0.748
18	−0.064	−0.04	0.745	−0.09	−0.041	0.747	−0.103	−0.042	0.747
19	-	-	-	−0.087	−0.039	0.747	−0.099	−0.041	0.747
20	-	-	-	−0.084	−0.033	0.747	−0.096	−0.034	0.747

**Table 4:** Calculated thermodynamic and kinetic desorption parameters (*T_D_* in K, *τ* in ns) and reversible storage metrics (N_A_, N_D_, N_P_, C_E_) for pristine and metal-decorated CQD at selected pressures and temperatures.

System	Li-CQD	Mg-CQD	Ti-CQD
*T_D_* (1 bar)	76.71	102.28	127.85
*T_D_* (3 bar)	87.28	116.37	145.46
*T_D_* (30 bar)	122.70	163.60	204.51
*T_D_* (100 bar)	155.76	207.68	259.61
*τ* (233 K)	0.03	0.06	0.15
*τ* (298 K)	0.02	0.03	0.10
*τ* (358 K)	0.01	0.02	0.04
*τ* (400 K)	0.01	0.01	0.02
C_T_ (wt%)	25.91	24.95	21.75
N_T_	18	20	20
N_A_	3.43	19.41	19.99
N_D_	0.06	2.75	17.66
N_P_	3.38	16.66	2.34
C_E_ (wt%)	6.16	21.7	3.1

**Table 5:** Comparative overview of predicted hydrogen storage performance in carbon-based nanomaterials, metal–organic frameworks (MOFs), covalent organic frameworks (COFs), and metal-decorated C_8_ quantum dots.

Material	Metal Decoration	${\overset{ˉ}{E}}_{\mathrm{ads}}$ (eV/H_2_)	wt%	Refs.
2D C_2_N layer	Li	≈−0.25	9	[22]
Graphene	Li	−0.15 to −0.25	6–8	[8]
Defected graphene	Li, Mg	−0.20 to −0.35	8–10	[27]
Carbon nanotubes	Ti	−0.40 to −0.60	4	[7]
C_60_ fullerene	Li	−0.20 to −0.30	7	[15]
Defected biphenylene	TM	−0.35 to −0.55	10	[21]
Irida-graphene	Sc	−0.18 to −0.45	21.6	[52]
R-graphyne-MOF	Li	−0.25 to −0.27	11.9	[53]
IRMOF-16	Mg	-	5.8	[54]
MOF-519	-	−0.12	10	[24]
IRMOF-10	Li	-	8.3	[55]
COF-1	Sc	−0.28	5.23	[56]
COF-1	Li	−0.26	7.7	[57]
Azatriphenylene-COF	Y	−0.45	6.4	[58]
CTF-1	Zr	−0.38	7.1	[59]
AzaCOF	Ti	−0.43	9.3	[60]
CQD	Li	−0.172	6.2	This work
CQD	Mg	−0.304	21.7	This work
CQD	Ti	−0.451	3.1	This work

## Data Availability

The data supporting the findings of this study are available within the article and its Supplementary Materials.

## Supplementary Materials

The following supporting information can be downloaded at: https://www.mdpi.com/article/10.3390/nano16050286/s1, Figure S1: Linear correlation between H_2_ adsorption energies calculated using ωB97X-3c/vDZP and ωB97X/def2-QZVP for representative C_8_-based systems, highlighting the minor basis-set effect; Figure S2: Effect of the exchange–correlation functional on H_2_ adsorption energies for C_8_-based systems; Table S1: Spin-state assessment for metal-decorated C_8_ systems using ωB97X-3c/vDZP. ΔE values are given relative to the lowest-energy spin state of each system; Table S2: Benchmark of H_2_ adsorption energies and key structural parameters for C_8_-based systems using different exchange–correlation functionals and basis sets.
