# Supplementary material for: Metal-Decorated C8 Quantum Dots as Lightweight Hydrogen Storage Materials: A Comprehensive DFT Study
Source: Nanomaterials (Basel). 2026 Feb 24;16(5):286. doi: 10.3390/nano16050286 (PMC12986565; doi:10.3390/nano16050286)
Supplement: Supplementary file 1 [file nanomaterials-16-00286-s001.zip › nanomaterials-4144921-supplementary.pdf]

Metal-Decorated C_8 Quantum Dots as Lightweight Hydrogen Storage Materials: A Comprehensive DFT Study

Seyfeddine Rahali[1,*], Ridha Ben Said[1], Youghourta Belhocine[2] , Suzan Makawi[1] and Bakheit Mustafa[1]

[1] Department of Chemistry, College of Science, Qassim University, Buraydah 51452, Saudi Arabia
[2] Laboratory of Catalysis, Bioprocess and Environment, Department of Process Engineering, Faculty of Technology, University of 20 August 1955, Skikda 21000, Algeria
* Correspondence: Seyfeddine Rahali: S.Rahali@qu.edu.sa

This Supporting Information provides additional validation of the computational methodology employed in this study. It includes (i) an assessment of spin-state stability for metal-decorated C_8 systems, and (ii) a benchmark analysis of H_2 adsorption energies with respect to basis-set size and exchange–correlation functional choice.

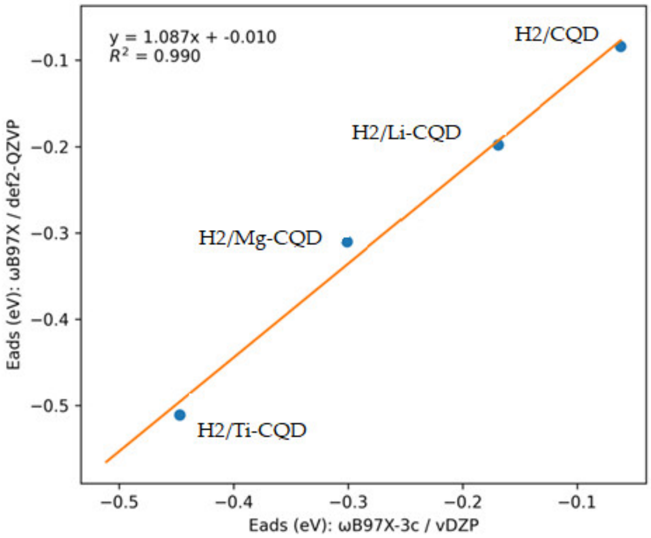

Figure S1. Linear correlation between H_2 adsorption energies calculated using ωB97X-3c/vDZP and ωB97X/def2-QZVP for representative C_8-based systems, highlighting the minor basis-set effect.

Figure S1 shows a linear correlation between H_2 adsorption energies calculated using the composite ωB97X-3c/vDZP approach and those obtained with the same underlying ωB97X functional and a larger def2-QZVP basis set. The excellent linear agreement (near-unity slope and high correlation coefficient) indicates that enlarging the basis set from double-ζ to quadruple-ζ induces only minor quantitative changes, confirming that basis-set incompleteness effects are small for the present systems.

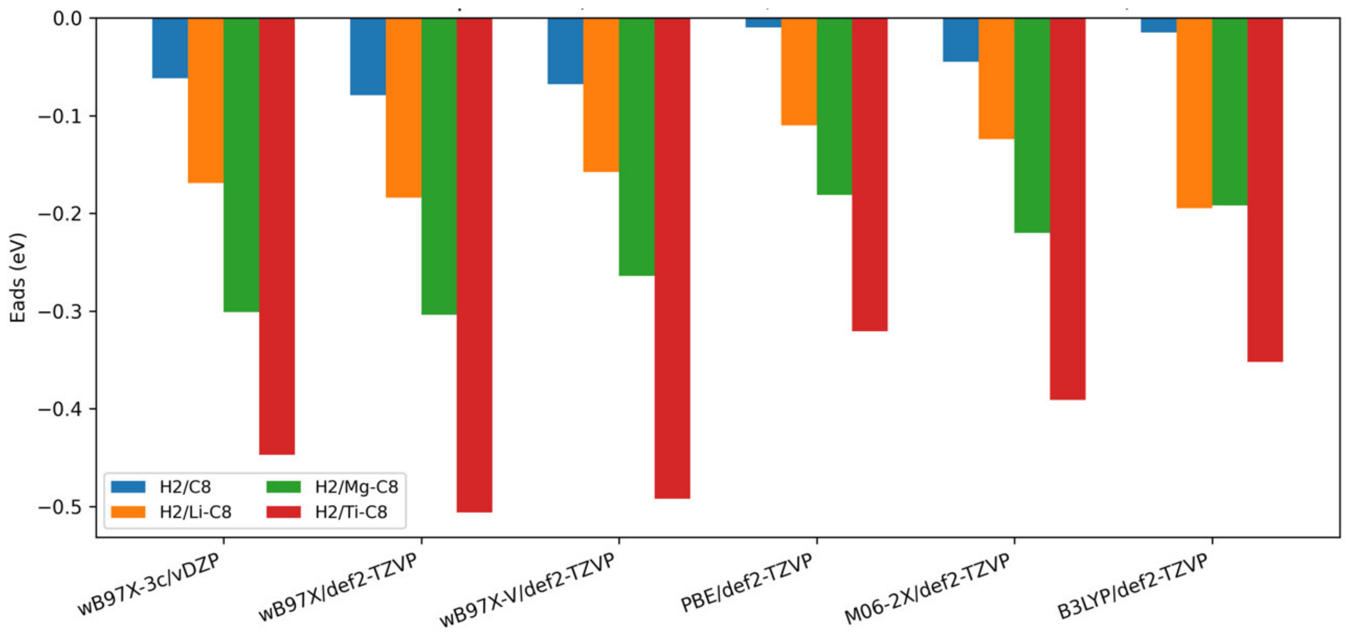

Figure S2. Effect of the exchange–correlation functional on H_2 adsorption energies for C_8-based systems.

Figure S2 illustrates the dependence of H_2 adsorption energies on the choice of exchange–correlation functional using a fixed def2-TZVP basis set. While absolute adsorption energies vary depending on the functional, dispersion-inclusive methods systematically predict stronger interactions than PBE. Importantly, all tested functionals preserve the same adsorption-strength ordering across C_8, Li–C_8, Mg–C_8, and Ti–C_8 systems.

Table S1. Spin-state assessment for metal-decorated C_8 systems using ωB97X-3c/vDZP. ΔE values are given relative to the lowest-energy spin state of each system.

System	Multiplicity	DE (ev)	⟨S²⟩
**Li–CQD**	2 (doublet)	0.000	0.771
	4 (quartet)	1.251	3.808
**Mg–CQD**	1 (singlet)	0.000	0.000
	3 (triplet)	0.196	2.029
**Ti–CQD**	1 (singlet)	1.253	0.000
	3 (triplet)	0.000	2.006
	5 (quintet)	1.528	6.049

Table S1 summarizes the relative energies of different spin states for metal-decorated C_8 systems calculated at the ωB97X-3c/vDZP level. The results confirm the stability of the selected ground-state multiplicities used throughout the study and indicate negligible spin contamination, as reflected by ⟨S²⟩ values close to their ideal expectations.

Table S2. Benchmark of H_2 adsorption energies and key structural parameters for C_8-based systems using different exchange–correlation functionals and basis sets.

Methods		ωB97X-3c vDZP	wB97X Def2-TZVP	wB97X Def2-QZVP	wB97X-V Def2-TZVP	wB97X-V Def2-QZVP	wB97M-V Def2-QZVP	PBE-D4 Def2-TZVP	M062X-D4 Def2-TZVP	B3LYP-D4 Def2-TZVP
H_2/CQD	C–C (Å)	1.46	1.46	1.46	1.47	1.47	1.47	1.48	1.46	1.47
	H….C (Å)	3.23	3.23	3.21	3.24	3.24	3.28	3.69	3.15	3.38
	E_{ads} (eV)	-0.062	-0.079	-0.084	-0.068	-0.067	-0.041	-0.010	-0.045	-0.015
H_2/Li-CQD	C–C (Å)	1.60	1.60	1.60	1.61	1.61	1.62	1.61	1.50	1.63
	Li–C (Å)	1.99	1.99	1.98	1.98	1.98	1.99	2.05	1.98	2.01
	H…Li (Å)	2.08	2.14	2.11	2.09	2.07	2.07	2.03	2.07	2.03
	E_{ads} (eV)	-0.172	-0.184	-0.198	-0.158	-0.183	-0.164	-0.11	-0.124	-0.195
H_2/Mg-CQD	C–C (Å)	1.63	1.63	1.64	1.65	1.65	1.65	1.62	1.63	1.64
	Mg–C (Å)	2.08	2.07	2.06	2.07	2.07	2.07	2.09	2.06	2.08
	H…Mg (Å)	2.19	2.19	2.15	2.18	2.18	2.21	2.25	2.15	2.21
	E_{ads} (eV)	-0.304	-0.304	-0.310	-0.264	-0.273	-0.241	-0.181	-0.220	-0.192
H_2/Ti-CQD	C–C (Å)	1.57	1.57	1.59	1.61	1.61	1.62	1.64	1.63	1.61
	Ti–C (Å)	2.04	2.02	2.02	2.03	2.03	2.03	1.99	2.08	2.03
	H…Ti (Å)	2.12	2.01	1.97	1.98	1.98	1.97	2.02	2.01	2.14
	E_{ads} (eV)	-0.451	-0.506	-0.511	-0.492	-0.507	-0.513	-0.321	-0.391	-0.352

Table S2 reports H_2 adsorption energies together with selected structural parameters obtained using different exchange–correlation functionals and basis sets. The data show that increasing the basis set from vDZP to def2-QZVP leads to only small changes in adsorption energies, whereas the choice of functional has a more pronounced effect on absolute values. Nevertheless, the relative adsorption trends remain consistent across all tested levels of theory.
